# Enhanced Delivery of Rituximab Into Brain and Lymph Nodes Using Timed-Release Nanocapsules in Non-Human Primates

**DOI:** 10.3389/fimmu.2019.03132

**Published:** 2020-01-23

**Authors:** Meng Qin, Lan Wang, Di Wu, Christopher K. Williams, Duo Xu, Emiko Kranz, Qi Guo, Jiaoqiong Guan, Harry V. Vinters, YooJin Lee, Yiming Xie, Yun Luo, Guibo Sun, Xiaobo Sun, Zhanlong He, Yunfeng Lu, Masakazu Kamata, Jing Wen, Irvin S. Y. Chen

**Affiliations:** ^1^Department of Microbiology, Immunology and Molecular Genetics, David Geffen School of Medicine at University of California, Los Angeles, Los Angeles, CA, United States; ^2^UCLA AIDS Institute, Los Angeles, CA, United States; ^3^Department of Chemical and Biomolecular Engineering, School of Engineering, UCLA, Los Angeles, CA, United States; ^4^Departments of Pathology & Laboratory Medicine (Neuropathology) and Neurology, David Geffen School of Medicine at UCLA, Los Angeles, CA, United States; ^5^Division of Hematology-Oncology, David Geffen School of Medicine at UCLA, Los Angeles, CA, United States; ^6^School of Nursing, UCLA, Los Angeles, CA, United States; ^7^Institute of Medical Biology, Peking Union Medical College, Chinese Academy of Medical Sciences, Kunming, China; ^8^Institute of Medicinal Plant Development, Peking Union Medical College, Chinese Academy of Medical Sciences, Beijing, China

**Keywords:** monoclonal antibody, central nervous system delivery, LNs delivery, non-human primate, nanocapsules

## Abstract

Tumor metastasis into the central nervous system (CNS) and lymph nodes (LNs) is a major obstacle for effective therapies. Therapeutic monoclonal antibodies (mAb) have revolutionized tumor treatment; however, their efficacy for treating metastatic tumors-particularly, CNS and LN metastases-is poor due to inefficient penetration into the CNS and LNs following intravenous injection. We recently reported an effective delivery of mAb to the CNS by encapsulating the anti-CD20 mAb rituximab (RTX) within a thin shell of polymer that contains the analogs of choline and acetylcholine receptors. This encapsulated RTX, denoted as n-RTX, eliminated lymphoma cells systemically in a xenografted humanized mouse model using an immunodeficient mouse as a recipient of human hematopoietic stem/progenitor cells and fetal thymus more effectively than native RTX; importantly, n-RTX showed notable anti-tumor effect on CNS metastases which is unable to show by native RTX. As an important step toward future clinical translation of this technology, we further analyzed the properties of n-RTX in immunocompetent animals, rats, and non-human primates (NHPs). Our results show that a single intravenous injection of n-RTX resulted in 10-fold greater levels in the CNS and 2-3-fold greater levels in the LNs of RTX, respectively, than the injection of native RTX in both rats and NHPs. In addition, we demonstrate the enhanced delivery and efficient B-cell depletion in lymphoid organs of NHPs with n-RTX. Moreover, detailed hematological analysis and liver enzyme activity tests indicate n-RTX treatment is safe in NHPs. As this nanocapsule platform can be universally applied to other therapeutic mAbs, it holds great promise for extending mAb therapy to poorly accessible body compartments.

## Introduction

Therapeutic monoclonal antibodies (mAbs) such as rituximab (RTX, anti-CD20 for B-cell lymphomas) and trastuzumab (anti-HER2 for breast cancer) have revolutionized treatment for various types of cancers. However, their benefit in treating metastasized tumors of the central nervous system (CNS) or through lymphatic vessels into lymph nodes (LNs) (1, 2), is transient and limited, increasing life expectancy by only a few months. A major mechanism that renders metastasis more resistant to mAb treatment than primary tumors is limited antibody delivery into the CNS and lymphatic vessels (3, 4). Intraventricular or intrathecal administration of mAbs allows for bypass of the blood-brain barrier (BBB), resulting in relative effectiveness of antibody therapy in treating brain tumor metastases (5, 6); however, neurotoxicity and rapid efflux are known to hinder mAb application for brain tumor treatment (7). Subcutaneous administration of mAb targeting metastatic tumors shows the advantages of entering lymphatic vessels and binding to metastases in lymph nodes (LNs) (8). However, the restriction to regional nodes, toxicity at injection sites, and limited reach to organs without lymphatic vessels are major obstacles to using subcutaneous administration of mAb in treating systemic metastases (9). Therefore, a systemic intravenous injection route is the ideal means for administration of mAb treatment against metastatic tumors.

To improve BBB penetration for mAb delivery to the brain, modifications with various chemicals or biological components, such as lipidation or molecular targeting ligands, have been attempted (10, 11). Another strategy uses colloidal carriers such as liposomes, micelles, and nanoparticles, which transport cargos across the BBB by endocytosis and/or transcytosis (12–14). Improved therapeutic efficacy of these approaches has been demonstrated in rodents with brain tumors, Alzheimer's disease, acute ischemic stroke, and Parkinson's disease (15–17); however, non-specific tissue accumulation—including in liver, spleen, and kidney—is known to mediate acute toxicity and further decrease the effective amount of mAbs in the CNS (18). Moreover, none of those approaches achieved improvement of both LN and brain delivery at the same time.

Our nanotechnology platform utilizes “nanocapsules” which form a thin polymer shell that encapsulates individual macromolecules, protein, RNA, or DNA inside and protects them from the physiological environment (19–27). The shell is formed by *in situ* polymerization of monomers and stabilized by environmentally-responsive crosslinkers; cargoes can be released only through cleavage of these crosslinkers. We tailored these nanocapsules for CNS delivery with zwitterionic properties imbued by polymer shells composed of 2-methacryloyloxyethyl phosphorylcholine (MPC), which is clinically approved for use in coatings on implanted medical devices. MPC renders the polymer shells of nanocapsules highly biocompatible and efficacious due to low protein adsorption, improved circulation times, and minimal immunogenicity (28, 29). Moreover, such nanocapsules can effectively penetrate the BBB and deliver encapsulated macromolecules to the CNS via nicotinic acetylcholine receptors and choline transporters (30). This technology has demonstrated efficacy for neural regeneration in mice with spinal cord injuries (31) and antibody therapies for primary brain tumors (32) in mice.

Rituximab (RTX), a chimeric anti-CD20 monoclonal antibody, is used for treatment of B-cell malignancies such as non-Hodgkin's lymphomas (NHL) as well as chronic lymphocytic leukemia (CLL) (33). RTX administration contributes significant advancements toward systemic CD20+ NHL control, but treatment of primary and relapsed CNS lymphomas is inefficient due to poor penetration through the BBB (4). We recently demonstrated clearance of human B-cell tumors with brain metastases in xenograft humanized NOD-SCID-IL2receptor γ^null^ (NSG) mouse models by RTX nanocapsules (n-RTX) (34). Though these results are promising, further studies are limited by the challenge in collecting successive samples of cerebrospinal fluid (CSF) from the same mouse for analysis; moreover, the delivery into LNs, which are highly atrophic, cannot be confirmed in NSG mice. To address these limitations, we designed studies of n-RTX in both rats and non-human primates (NHPs) to further investigate delivery and biodistribution in both lymphatic tissues and CNS, and B-cell ablation in NHPs. Following a single IV dose of n-RTX, encapsulated RTX is released and maintained in blood for weeks resulting in effective B-cell ablation in blood and lymphatic tissues of NHPs. Importantly, we show significantly improved RTX delivery to the CNS and lymph nodes with no notable adverse effects.

## Results

### Formulation of Nanocapsules With Hydrolysable Crosslinkers to Release mAbs

**Figure 1:**
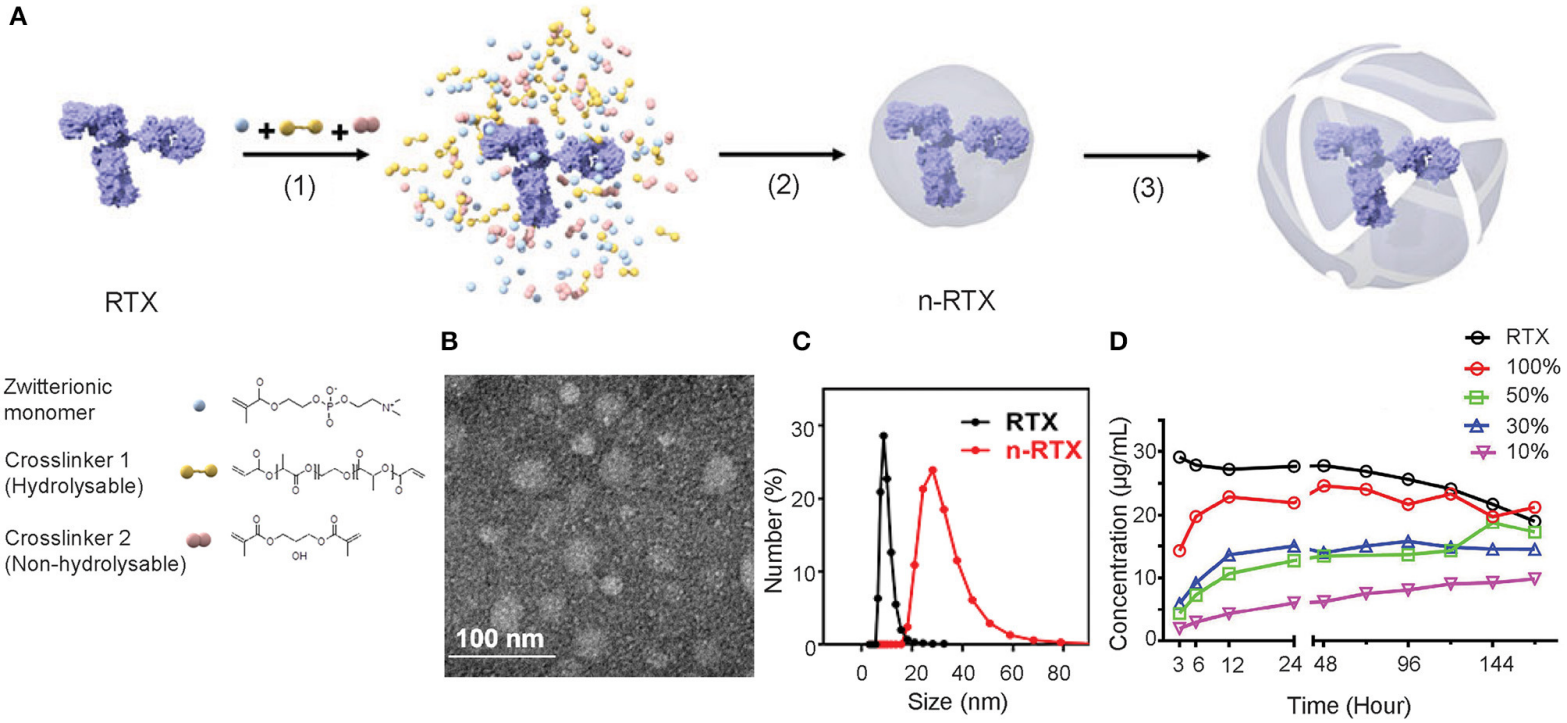
Developing timed-release nanocapsules of RTX. **(A)** Scheme of the synthesis of and release by timed-release RTX nanocapsules (n-RTX) by (1) enriching zwitterionic monomer (MPC), hydrolysable crosslinker 1 (PLA-PEG-PLA) and non-hydrolysable crosslinker 2 (GDMA) around a RTX molecule, (2) *in-situ* polymerization of the monomer and crosslinkers forming a thin shell of polymer around an RTX molecule, and (3) releasing RTX when polymer shells are degraded under physiological condition. **(B)** Transmission Electron Microscopy image of n-RTX. **(C)** Size distribution of n-RTX measured by dynamic light scattering measurement. **(D)** Nanocapsules with mixed hydrolysable crosslinkers achieve timed release of RTX in rhesus macaque plasma. The nanocapsules synthesized with mixed PLA-PEG-PLA and GDMA crosslinkers at different ratios. Release kinetics of n-RTX with 100, 50, 30, 10, and 0% of PLA-PEG-PLA were tested. Thirty micrograms of nanocapsules were incubated in 1 mL of rhesus macaque plasma at 37°C. The concentration of released RTX was determined by ELISA.

A formulation of nanocapsules with timed-release capabilities *in vivo* was synthesized based on previously published nanocapsules (19). We screened and selected two crosslinkers to sustain release at physiological conditions *in vivo*: hydrolysable crosslinker—poly (lactide-co-glycolide)-b-poly(ethylene glycol)-b-poly(lactide-co-glycolide) (PLA-PEG-PLA) and non-hydrolysable crosslinker—glycerol dimethacrylate (GDMA), which degrade rapidly and slowly, respectively, at physiological pH conditions. The ratios between and GDMA impact the release kinetics. With a higher PLA-PEG-PLA ratio, the crosslinkers will degrade in a shorter time. As the crosslinkers degrade, the shells of nanocapsules will loosen, swell, and dissociate resulting in the release of encapsulated mAbs. As shown in Figure 1A, nanocapsules encapsulating mAbs are synthesized through the following processes: first, the zwitterionic monomer (MPC) and two crosslinkers, PLA-PEG-PLA and GDMA, are enriched around the surface of the mAb (in this case, RTX) through hydrogen bonding (Step 1). Subsequent polymerization in an aqueous solution at 4°C wraps each molecule with a thin shell of polymer through *in situ* free-radical polymerization (Step 2). Finally, crosslinkers stabilize the polymer structure and release mAbs upon hydrolysis (Step 3). Transmission electron microscopy (TEM) and dynamic light scattering (DLS) measurements show that these nanocapsules form a spherical morphology of 20–30 nm encasing mAb molecules inside (Figures 1B,C). Dependent on the ratios between PLA-PEG-PLA and GDMA, nanocapsules release RTX at different rates when incubated in rhesus macaque plasma *in vitro* (Figure 1D). The RTX concentration was detected by enzyme-linked immunosorbent assay (ELISA) using anti-RTX (anti-idiotype) antibody, which can only detect the free RTX released from nanocapsules since nanocapsules shields the epitopes of encapsulated antibodies by the polymer shells. The n-RTX with 50 and 30% PLA-PEG-PLA crosslinkers showed an intermediate level of release over 6 days. We already demonstrated improved CNS delivery of n-RTX with 50% PLA nanocapsules in mice in our published work (35), so the same formulation for following *in vivo* studies in rats and NHPs was used. We also demonstrated that improvement of CNS delivery with this nanocapsules formulation is applicable to other therapeutic mAbs. To prove this point, we tested Herceptin (anti-Her2) for breast cancer; similarly to n-RTX, Herceptin nanocapsules (n-Hercepin) show increased delivery to the CNS (Figure S1).

### Improved Delivery to CNS and LNs of Rats by RTX Nanocapsules

**Figure 2:**
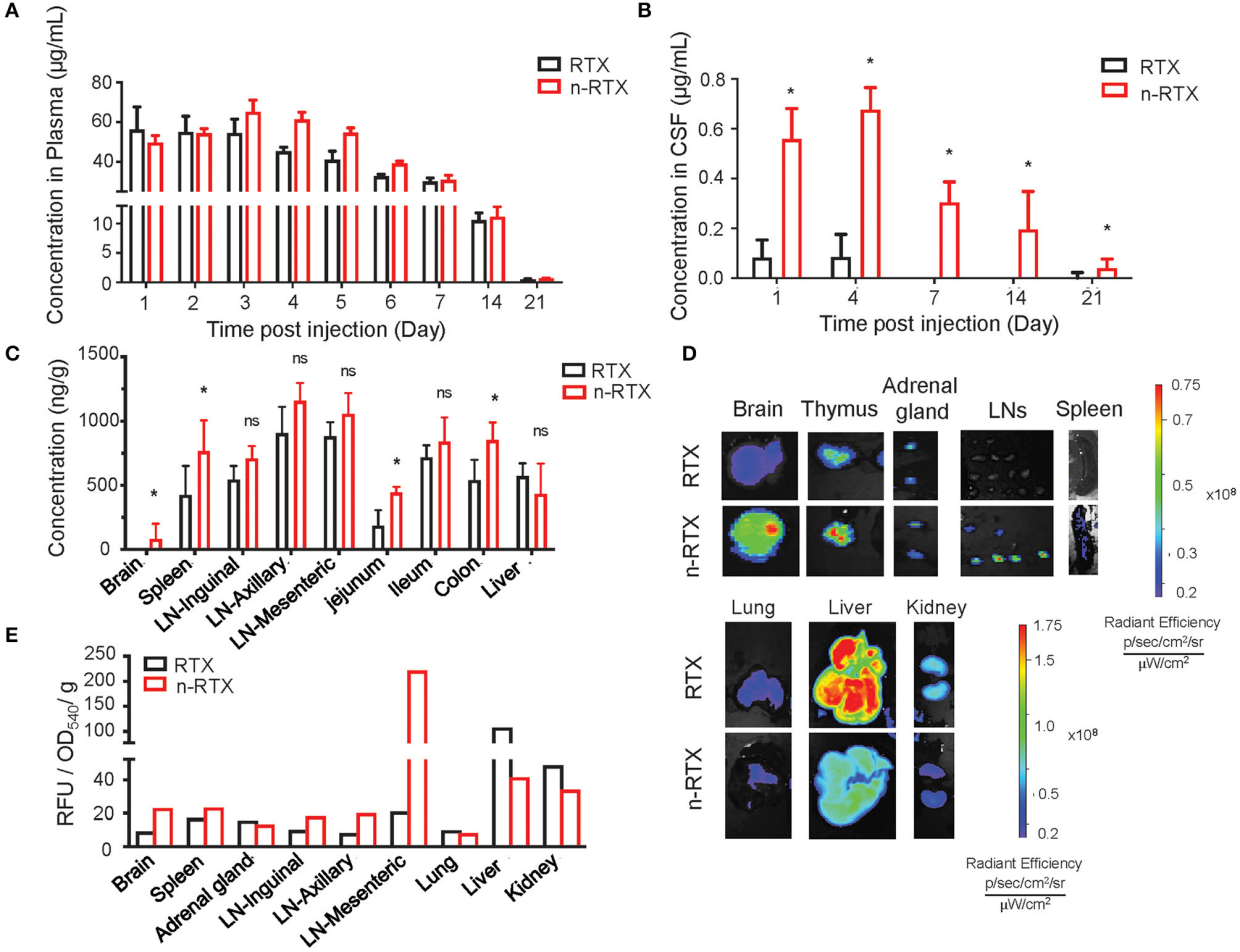
Nanocapsulation of RTX improves levels of its tissue penetration, including CNS, in rats. A single dose (5 mg/kg) of native RTX or n-RTX was administered in rats through IV (intravenous) injection (*n* = 3). **(A–C)** The concentrations of free RTX in plasma **(A)** on Days 1, 2, 3, 4, 5, 6, 7, 14, and 21 and CSF **(B)** on Days 1, 4, 7, 14, and 21 were determined by ELISA. **(C)** Tissues, including LNs at three different locations, jejunum, ileum, colon, spleen, liver, and brain, were harvest at necropsy after perfusion on Day 22. Tissues were homogenized in PBS (1 mg tissue in 100 μl PBS), and tested by ELISA for RTX concentrations. A higher single dose (15 mg/kg) of fluorescent labeled native RTX or n-RTX was administered in rats through IV injection (*n* = 1) and monitored on IVIS *in vivo* imaging **(D,E)**. Organs were isolated and monitored by IVIS imaging on Day7 after injection. **(E)** Signal intensity from free RTX in each organ was quantified by software Living Image. Data were generated from *n* = 3 rats. Data are shown as means ± s.d. of biological triplicate. Statistical significance between native and nanocapsules was determined by one-tailed Mann–Whitney–Wilcoxon test. *Significant, *p* < 0.1; ns, not significant.

We demonstrated the improved delivery to CNS and LNs by n-RTX using rats where greater amounts of CSF facilitate serial sampling within the same animals and mature LN formation enables clear visualization and detection in the lymphatic system. Native RTX and n-RTX, normalized with of the same amount of RTX for a single IV dose, showed comparable RTX level in plasma (Figure 2A). Whereas, native RTX in CSF was <0.1% of plasma levels, RTX released from n-RTX was 1%, and was maintained this level to days 14 and 21 when native antibody was barely detectable at day 4 (Figure 2B). At the endpoint, RTX levels in brain tissue were consistent with the CSF results; higher levels of RTX were observed in the n-RTX treated animals (Figure 2C). These results provide proof of concept for improved antibody delivery in the CNS compared to the native form. Improved levels of RTX from n-RTX are also observed in lymphatic-tissues, which include organs such as spleen, LNs in different locations, and three sections of the small intestine (Figure 2C). Tissue imaging shows clear biodistribution of fluorescently labeled native RTX and n-RTX on day 1 post-injection (Figure 2D). Compared to native RTX, n-RTX shows an improved distribution in lymphatic-tissues containing organs including LNs and spleen and, importantly, reduced accumulation in lung, liver, and kidney—major organs involved with clearance of recombinant antibodies. The decreased accumulation in lung, liver, and kidney is due to the superior anti-fouling property of n-RTX, which decrease the uptake by immune surveillance cells. The difference in biodistribution between RTX and n-RTX is further confirmed by quantifying the relative fluorescence units of the fluorescence label from each organ (Figure 2E).

### Enhanced Delivery of RTX Nanocapsules to the Brain of Rhesus Macaques

**Figure 3:**
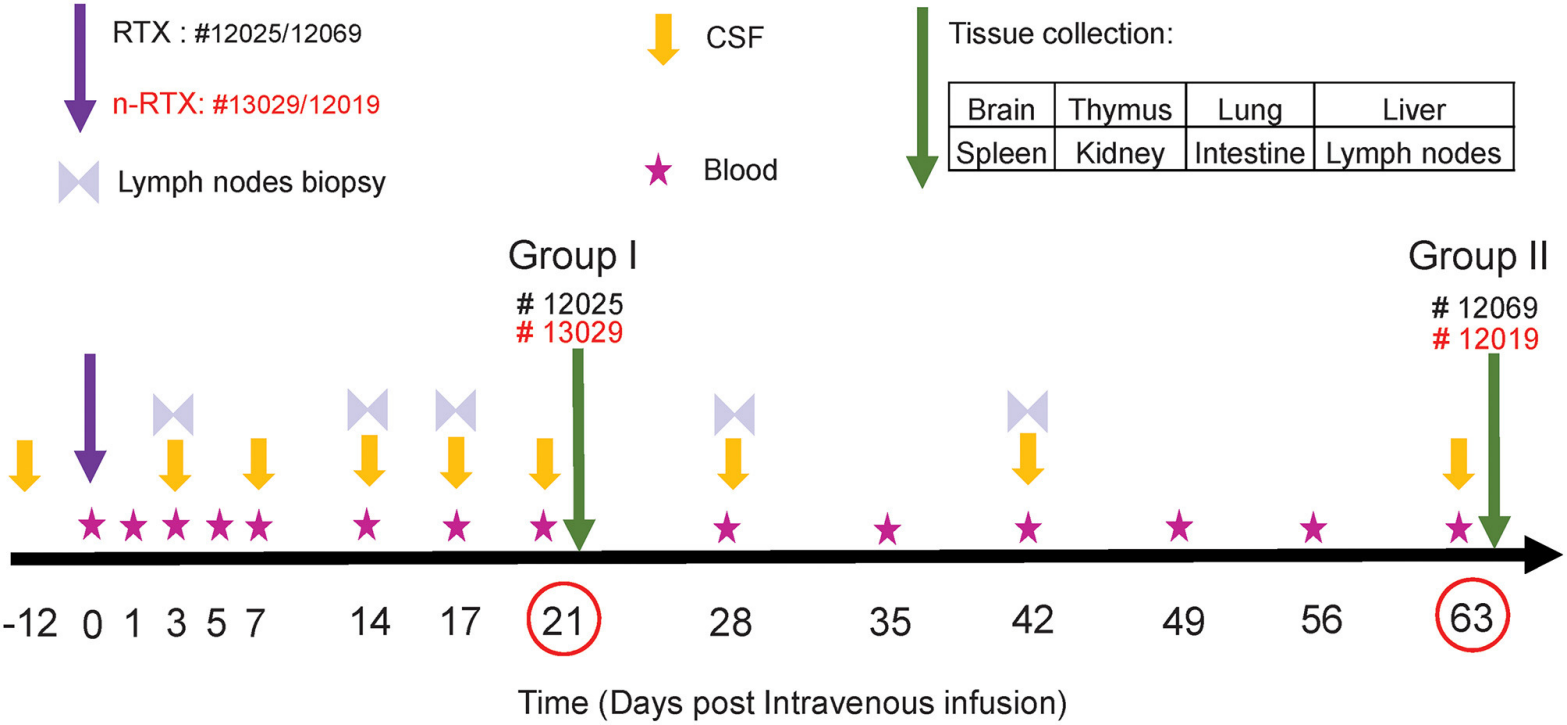
Experimental schedule of RTX brain delivery in non-human primates. Four rhesus macaques were administered with a single 5 mg/kg dose of native RTX (Monkey ID #12025 and #12069) and n-RTX (Monkey ID #13029 and 12019) via IV infusion. Peripheral blood, CSF, and Inguinal lymph nodes were collected as scheduled. Other tissues were harvested at necropsy on Days 21 or 63 after perfusion.

As an essential step toward future clinical translation of nanocapsules, we further analyzed the properties of n-RTX in non-human primates (NHPs). The study design is summarized in Figure 3. Four rhesus macaques were intravenously infused with a single dose (5 mg/kg, normalized for the RTX amount) of native RTX or n-RTX and processed for necropsy on Day 21 (Group I, #12025 and #13029) or Day 63 (Group II, #12069 and 12019). Blood samples were collected on Days 1, 3, 5, and 7 in the first week, and every 7 days after that (Figure 3, Stars). CSF sample collection started before infusion as a baseline (Figure 3, Day-12), on Day 3 post-infusion, and continued every 7 days until Day 21 for Group I; for Group II, CSF collection was initiated on Day 1 post-infusion and continued every week until Day 63. Lymph node (LN) biopsies were performed on Days 3 and 14 in Group I and on Days 3, 17, 28, and 42 in Group II (Figure 3, Double triangles). Eight tissues, including brain, thymus, lung, liver, spleen, kidney, intestine, and LNs, were harvested at necropsy following perfusion. LNs from different locations (inguinal LN, axillary LN, and mesenteric LN), and three pieces (3 × 3 cm) from each of other tissues were collected and used for ELISA assays. Brain and LN samples were also used for immunohistochemical (IHC) analysis.

**Figure 4:**
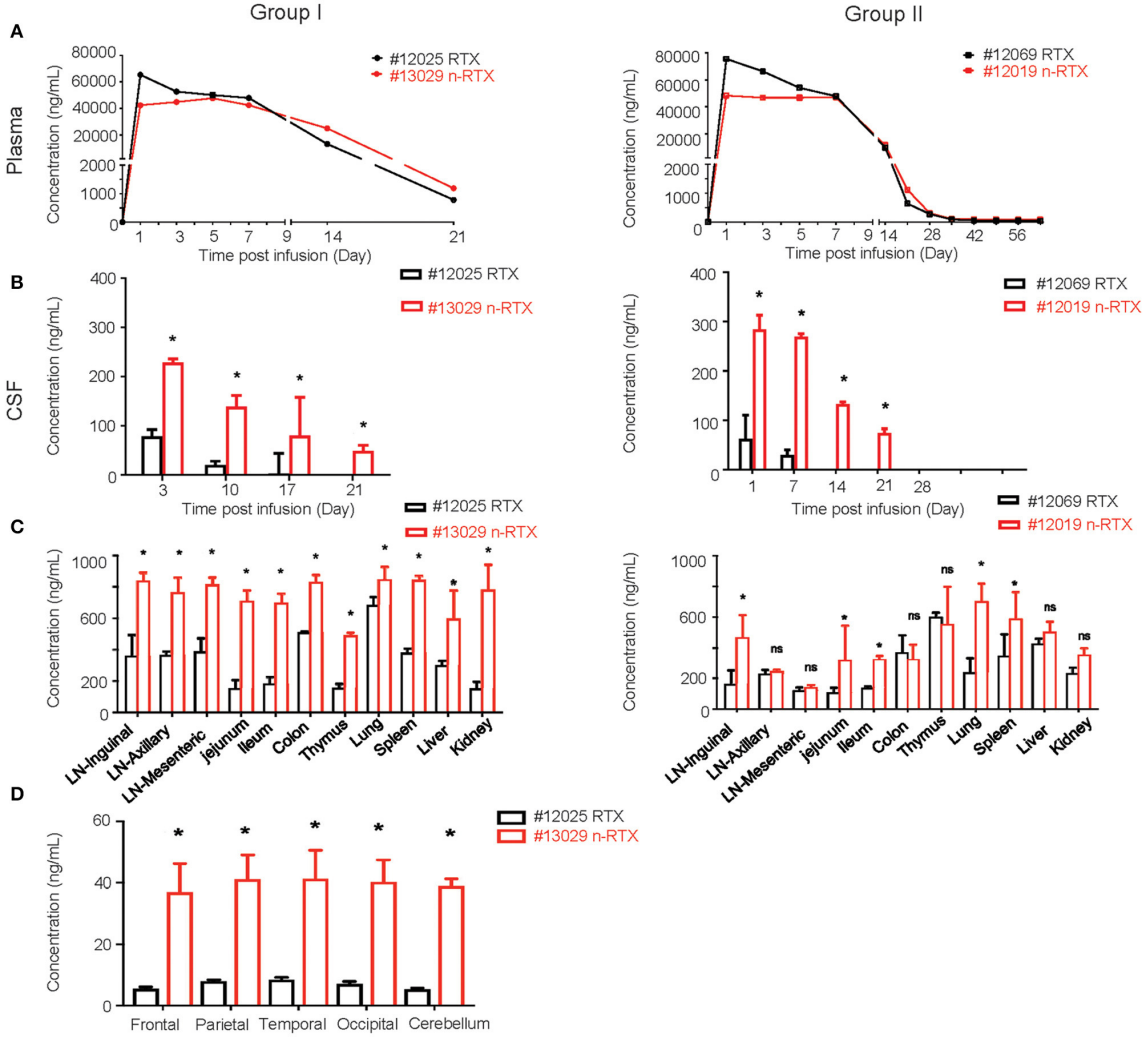
Nanocapsulation of RTX improves levels of its tissue penetration in non-human primates. A single dose (5 mg/kg) of native RTX or n-RTX was administered in rhesus macaques through IV infusion **(A,B)**. The concentrations of free RTX in plasma **(A)** and CSF **(B)** were determined by ELISA. **(C)** At the endpoint of Group I (Day 21) and Group II (Day 63), LNs at three different locations, jejunum, ileum, colon, thymus, lung, spleen, liver, kidney, and brain were harvest after perfusion. Whole LNs and three pieces (3 × 3 cm) of other tissues were homogenized in PBS (1 mg tissue in 100 μl PBS) for ELISA test. **(D)** Five different regions of the brain (frontal, parietal, temporal occipital lobes, and cerebellum) were homogenized in PBS (1 mg tissue in 100 μl PBS) for ELISA test. Data are shown as means ± s.d. of biological triplicate. Statistical significance between native and nanocapsules was determined by one-tailed Mann–Whitney–Wilcoxon test. *Significant, *p* < 0.1, ns, not significant.

We successfully demonstrated the enhanced CNS delivery of n-RTX in rhesus macaques (Figure 4). The levels of RTX released from n-RTX in rhesus macaque plasma was slightly lower on Day 1 compared to that of native RTX, probably due to the controlled RTX release from n-RTX in plasma (Figure 4A). The plasma level remained stable for 1 week, followed by a gradual decreased over time. Two animals treated with n-RTX (#13029 and 12019) showed 5- and 2.5-fold greater levels of free RTX in the CSF compared to those in native RTX-treated animals (#12025 and 12069), respectively, within the first week (Figure 4B). Native RTX fell below detection limits by Day 14, whereas free RTX released from n-RTX persisted until Day 21 in both groups. RTX concentration in the tissue homogenates was also assessed by ELISA (Figure 4C). In Group I, the animal treated with n-RTX (#13029) showed significantly higher levels of RTX in all tissues than that treated with native RTX (#12025) in all tissues. A similar trend was confirmed with animals in Group II, but the difference between those two treatments was less significant. Five regions of the brain, including the frontal, parietal, temporal occipital lobes, as well as cerebellum, were homogenized separately for ELISA. RTX was only detectable in Group I. RTX released from n-RTX was observed at approximately 4-fold levels in all brain regions than that in the brain tissues from the RTX-treated animal (Figure 4D).

**Figure 5:**
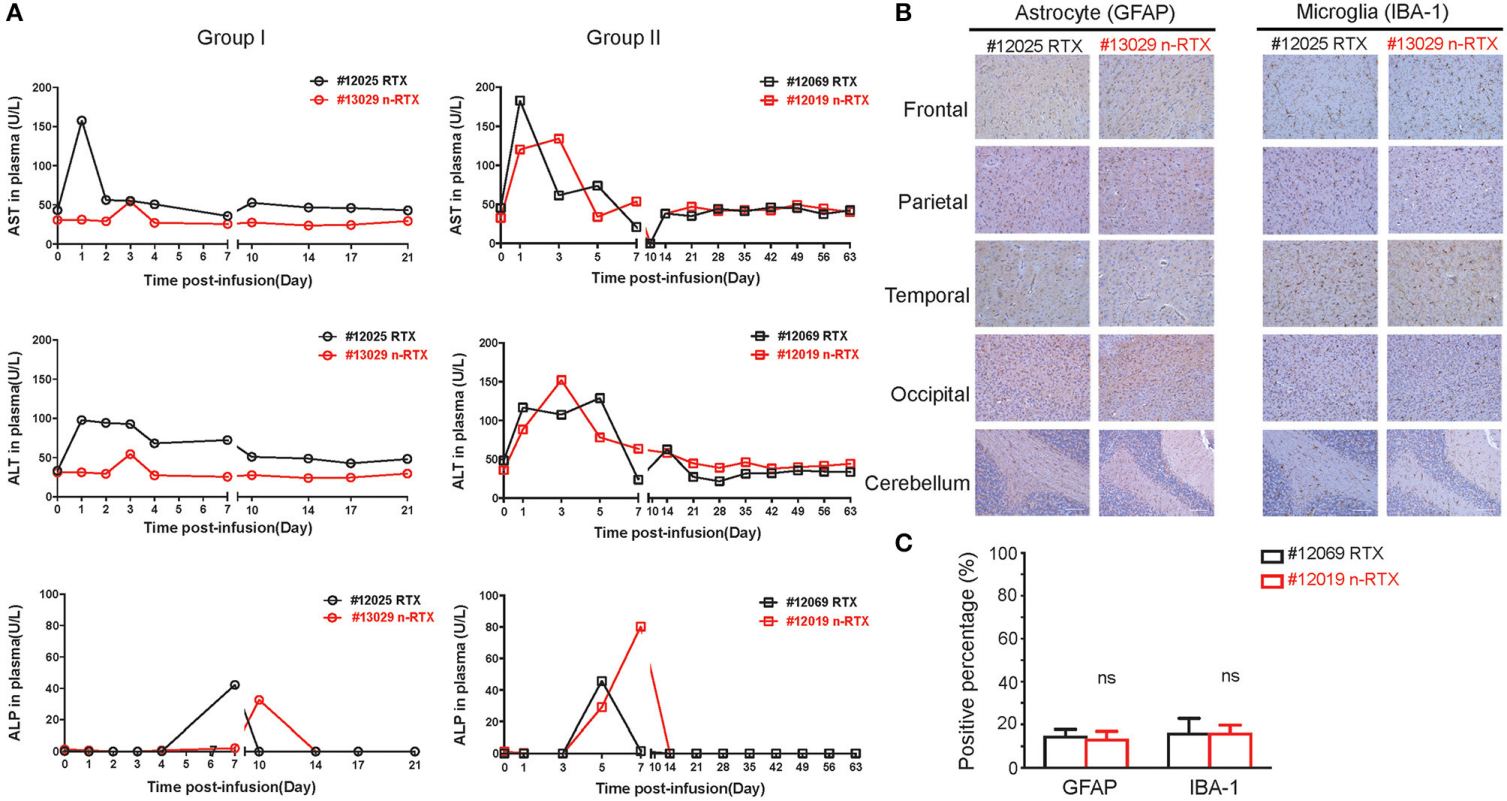
RTX nanocapsules do not mediate notable levels of acute toxicity in the liver and brain. **(A)** Rhesus macaques were administrated with a single dose (5 mg/kg) of native RTX or n-RTX. Plasma samples were collected as scheduled and served for measuring levels of aspartate transaminase (AST), alanine transaminase (ALT), and alkaline phosphatase (ALP) by ELISA. **(B)** Brain tissues obtained from five different parts were fixed by 4% paraformaldehyde and processed for IHC analysis. Representative images of immunohistochemical staining for GFAP (astrocyte marker) and IBA-1 (microglial marker) were shown. Scale bar = 100 μm. **(C)** A positive pixel count in images (4 mm^2^) from five different brain parts was quantified using Aperio ScanScope slide scanner. Positive pixel calculation for GFAP used the following variables: hue width = 0.371 and color saturation threshold = 9.7e-002. Positive pixel calculation for IBA-1 used the following variables: hue width = 0.5 and color saturation threshold = 4.0e-007. % positivity was calculated by total number of positive pixels divided by the total number of pixels. Data are shown as means ± s.d. of biological triplicate. Statistical significance, compared with the native RTX group, was determined by one-tailed *t*-test with Welch's correction. ns, not significant.

Liver toxicity is a major concern for use of nanomedicines *in vivo* (35). Thus, liver damage is the one of the indexes of nanomedicine safety. In order to show that this nanocapsule platform improves brain delivery efficiency of therapeutic antibodies through systemic injection without inducing liver toxicity, three liver enzymes indicating acute liver toxicity were closely measured over the course of experiments: alanine aminotransferase (ALT), aspartate aminotransferase (AST), and alkaline phosphatase (ALP) (Figure 5A). As we confirmed in mice previously (34), no notable differences exist in levels between animals treated with RTX or n-RTX. Neither a complete liver function assay including serum globulin (GLB), indirect bilirubin (IBIL), gamma-glutamyl transferase (γ-GT), total protein (TP), and albumin (ALB), nor a blood chemistry test for white blood cell (WBC) count, lymphocyte count, monocyte count, neutrophil count, hemoglobin (HGB), hematocrit (HCT), and platelet counts (PLT), showed major differences between those animals (Figure S2). We have hereby concluded that n-RTX increases antibody delivery into the CNS by 4–10-fold with no detectable level of acute systemic toxicity.

We further showed no potential neurotoxicity caused by n-RTX treatment. Two well-known neurotoxicity markers were used in this test: glial fibrillary acidic protein (GFAP), of which levels increase with reactive gliosis (36), and ionized calcium-binding adapter molecule (IBA-1), of which levels increase upon mediating neuroinflammation (37). Compared to native RTX-treated animals, brain tissues of n-RTX treated animals showed normal morphology and no elevated expressions of those two markers (Figures 5B,C).

### Comparison of Effector Activity Mediated by Native RTX and n-RTX

**Figure 6:**
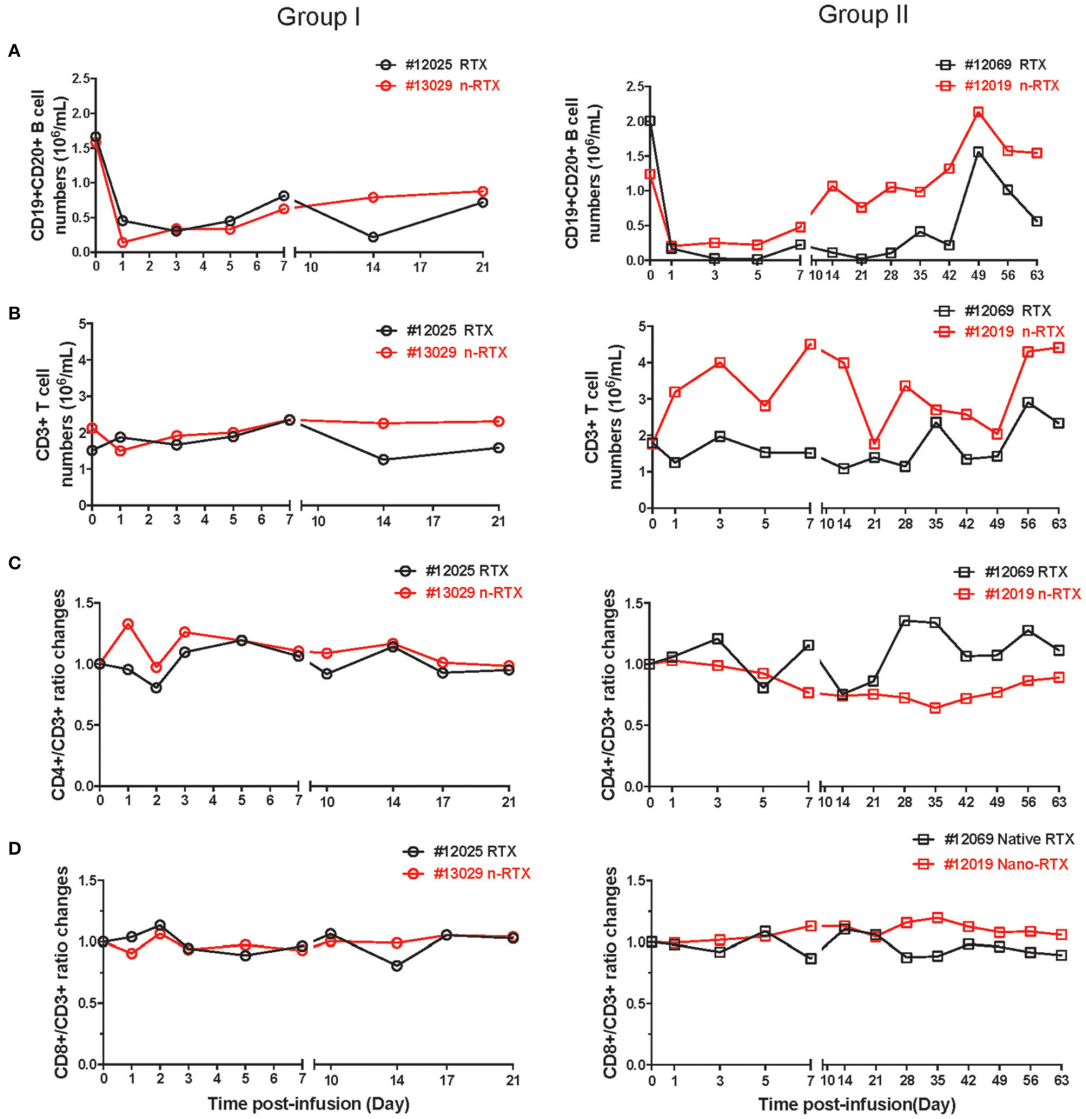
Both native and nanocapsulated RTXs mediate similar levels of peripheral blood B cell depletion in rhesus macaques. Whole blood was collected as scheduled and processed for monitoring immunophenotyping by flow cytometry. **(A)** CD19+CD20+ B cells, **(B)** CD3+ total T cells, **(C)** CD4+T cells, and **(D)** CD8+T cells. **(A,B)** CD19+CD20 B cells and CD3+ T cells were showed by absolute numbers per 1 mL of blood. **(C,D)** The percentages of CD4+ T cells and CD8+ T cells in CD3+ T cells were normalized over baseline.

Lastly, we observed comparable B cell depletion by RTX and n-RTX in peripheral blood of NHPs. CD20 can be internalized by the binding of RTX (38, 39), so single staining for cell surface CD20 may underestimate total B cell levels. To more accurately measure B cell levels, we stained cells with both CD19 and CD20 antibodies. To minimize CD20 epitope masking by RTX, we used the CD20 antibody clone L26, which recognizes different epitope on rhesus CD20 molecules not blocked by RTX (40). Analysis by single staining for CD19+ showed similar results to double staining for CD19+/CD20+ cells (data not shown). CD19+/CD20+ B cell levels dropped ~80–90% within the first 7 days after treatment by both RTX and n-RTX (Figure 6A). Aside from the expected drop in B cells, the numbers of total WBCs, neutrophils, lymphocytes, and monocytes were relatively stable (Figure S3), indicating B-cell specific depletion by RTX in both forms. Levels of total CD3+ cells, CD3+/CD4+ T cells, and CD3+/CD8+ T cells were also stable over the course of treatment (Figures 6B–D).

**Figure 7:**
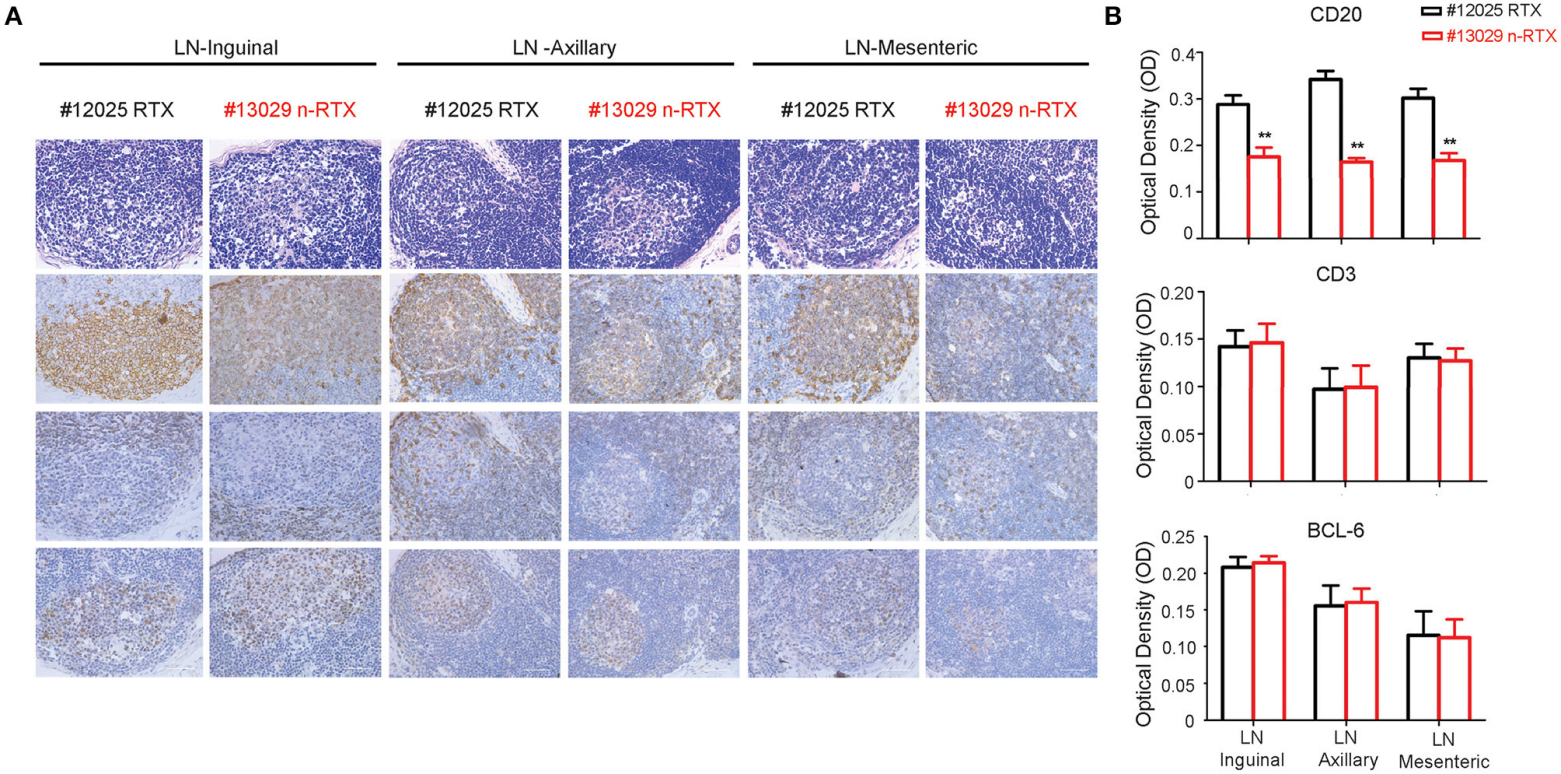
Nanocapsulated RTX mediates superior B-cell depletion in LNs of rhesus macaques. LNs from three different locations (inguinal, axillary, and mesenteric) were collected at necropsy on Day 21 from Group I rhesus macaques with a single dose (5 mg/kg) of native RTX or n-RTX via IV and processed for immunohistochemical (IHC) analyses. **(A)** H&E (top) and IHC staining of CD20 (B cell, second), CD3 (T cell, third), and Bcl-6 (Germinal center, bottom) were performed on serial sections. Panels from left to right show the expression level in axillary, mesentery, and inguinal LNs, respectively. Scale bar = 50 μm. **(B)** 6.8 × 5 cm of each CD20 and CD3 positive staining (*n* = 7), and 3.4 × 4.2 cm of Bcl-6 (*n* = 4) were randomly selected and gray pixel values were measured by Image J software. Each bar indicates the mean inverse gray value ± standard deviation (mean ± SD). One-tailed *t*-test with Welch's correction was used for the statistical analysis. **p < 0.01. H&E, hematoxylin and eosin stain.

To assess the specificity of depletion and accessibility to B cells in lymphoid tissues, we analyzed levels of B-cell depletion in LNs at three different localities: axillary, mesenteric and inguinal, by IHC analysis. Antibodies for CD20+, CD3+, and Bcl-6 were included to distinguish three different types of cells, B cells, T cells, and T follicular helper cells (Figure 7A). By further quantitative analysis of IHC staining results, CD20+ B cells showed a lower density in all three LNs of animals treated with n-RTX compared to that of native RTX on Day 21 (Figure 7B). Ratios of B cell against T cell (B/T ratio) detected by flow cytometry in inguinal LN indicated that n-RTX mediated prolonged B cell depletion over the course of treatment compared to native RTX (Figure S4). In contrast, no significant difference in levels of CD3+ T cells was confirmed between LNs treated with native RTX or n-RTX. The level of Bcl-6 expression in LNs, a marker of germinal center B cells and T follicular helper cells, showed no difference between two animals.

## Discussion

We utilized a platform wherein single mAbs are encapsulated within a thin polymer shell, called a “nanocapsule,” to improve delivery of anti-tumor antibodies to the brain. Formulations were modified to utilize neutral polymers with zwitterionic properties (MPC polymers) and crosslinkers that hold the shell together which are later gradually hydrolyzed for timed release of mAb in a suitable microenvironment. This successful nanocapsule design sustains RTX in circulation, allows penetration into the brain, reaches deep tissues including LNs, and releases RTX in a controlled fashion. Our results detail the first test of a zwitterionic nanocarrier—nanocapsule—for therapeutic mAb delivery in NHPs. Importantly, the data clearly show efficient and prolonged delivery of RTX into both the CNS and LNs by a single dose of intravenous injection with no notable acute liver toxicity nor neurotoxicities.

Therapeutic mAbs are effective for treating several tumors; however, their performance is limited in the treatment of metastases in CNS and LNs, where the physical barriers are considered as one of the major obstacles to achieve effective mAbs delivery. Although subcutaneous administration is considered as an effective route for LN delivery through the absorption by lymphatic capillaries, the delivery into the lymphatic system is delayed due to the limitation of the absorption rate (41, 42). Moreover, the brain delivery of mAbs through subcutaneous administration is challenging due to the low concentration of mAbs in plasma. Therefore, intravenous injection is still a commonly used administration route for mAb therapy. However, achieving a high plasma concentration is insufficient for treatment of brain cancers due to poor delivery through the BBB. While multiple strategies have been attempted to facilitate greater antibody penetration through the BBB, the potential risks outweigh most benefits. One approach is to disrupt the tight junctions, allowing for passage, however, this approach raises safety concerns for the brain environment (43). Other approaches are the modification and engineering of mAbs to improve brain delivery; attaching charged or lipophilic moieties to mAbs to enhance absorptive-mediated transport, but those modifications are known to result in unexpected biodistribution, low sustainability, and insufficient efficacy (44, 45). mAbs have also been encapsulated or conjugated with nanoparticles or liposomes to induce receptor-mediated transcytosis on the BBB (46, 47), but the efficacy, safety, and stability in NHPs are still unknown. Thus, despite decades of effort, effective delivery of mAbs to the both LNs and the CNS remains challenging.

We used the nanocapsule platform to achieve systemic delivery of RTX into both the CNS and LNs in both rodents and NHPs with single intravenous injection. The choline and acetylcholine analogous structures on the MPC polymer chains of nanocapsules, which can induce transcytosis through the BBB via nicotinic acetylcholine receptors and choline transporters, play an important role to accomplish CNS penetration (30). In NHPs treated with nanoencapsulated RTX, levels of RTX in the CSF were 100 to 300 ng/mL over 14 days, which are 4–10-fold greater than that observed for native RTX in the CSF. It indicates that our nanocapsule platform achieved higher brain delivery compared to animals treated with native RTX. Moreover, similar to animals treated with native RTX, those treated with nanocapsules exhibited no notable blood, liver, or neuronal toxicities. These studies were conducted in immune competent rats and outbred NHP more closely modeling human therapy than in our previous studies utilizing immunodeficient mice. While these levels of RTX in the CNS were sufficient to clear xenografted B cell lymphomas in the immunodeficient mice, it is unclear whether these levels are high enough to have therapeutic efficacy in humans. Our results on biodistribution and safety profiles provide a rationale for further assessing efficacy and safety in human clinical studies.

## Conclusion

Our studies suggest a non-invasive facile solution suitable for human application to address this critical issue of poor anti-cancer mAb deliverance to both LNs and the CNS. To date, similar results are obtained with one additional clinically approved anti-cancer mAb, anti-Her2 (Herceptin), as well as other non-therapeutic mAbs such as anti-CD4 (OKT4) (unpublished data). The platform is highly versatile, with biodistribution and pharmacokinetics being readily adjustable by rational choice of chemical formulation of the polymer shell. In addition to timed-release functionality via hydrolysis of crosslinkers under physiological pH conditions, mAb release can be further controlled by crosslinkers sensitive to environmental factors such as endosomal low pH and proteases (21). Our study supports the translation of therapy from animals to human clinical studies. Further enhancements of the platform may produce therapeutic delivery options for other diseases requiring mAbs delivery into the CNS and/or LNs.

## Materials and Methods

### Materials

All chemicals were purchased from Sigma-Aldrich (St. Louis, MO, USA) unless otherwise noted. All cell culture reagents were purchased from ThermoFisher Scientific (Waltham, MA) unless otherwise noted. Hydrolysable crosslinker Poly(DL-lactide)-b-Poly(ethylene glycol)-b-Poly(DL-lactide)-diacrylate triblock (PLA-PEG-PLA) was purchased from PolySciTech Akina, Inc. (West Lafeyette, IN). Capture antibody for ELISA against rituximab was purchased from Bio-Rad Laboratories (HCA0620, Hercules, CA). Anti-CD19, anti-CD20, anti-CD3, anti-CD4, and anti-CD8 for flow cytometry were purchased from BioLegend, Inc (San Diego, CA, USA). Antibodies for IHC staining were purchased from different vendors, identifying separately in Immunohistochemistry section. Rituximab, RITUXAN (Genentech, Inc.) was purchased from the UCLA hospital pharmacy.

### Synthesis of Nanocapsules

The mAbs (RTX and Herceptin) were encapsulated via *in-situ* polymerization technology. The mAb solution (2.2 mg/mL in PBS) was mixed with MPC as monomer (40% m/v in PBS) and AI102 (PLA-PEG-PLA, 10% m/v in PBS) as well as glycerol dimethacrylate (GDMA, 10% m/v in DMSO) as degradable crosslinkers. Then the polymerization was initiated by adding ammonium persulfate (APS, 10% m/v in PBS) and N, N, N′, N′-tetramethylethylenediamine (TEMED) solution. Synthesized nanocapsules were dialyzed against PBS and purified by passing through a hydrophobic interaction column (Phenyl-Sepharose 4BCL). Since the nanocapsules possess a super-hydrophilic surface, their binding affinity to the column is much weaker than the native mAb. Thus, encapsulated protein will be eluted out with a high salt concentration buffer (10xPBS), whereas the native mAb binds on the column. Purification is confirmed by DLS and ELISA to show no free mAbs. Detailed parameters of the synthesis are provided in the Table S1.

### Transmission Electron Microscopy (TEM) and Dynamic Light Scattering (DLS) Measurements of the Nanocapsules

The nanocapsule solution (0.2 mg/mL) 10 μL was dropped onto a carbon–coated copper grid. After 45 s incubation, excess amount of the samples was removed. The samples are stained with 1% phosphotungstic acid (PTA) at pH 7.0 after being rinsed with distilled water for three times. To investigate the size and zeta potential of the nanocapsules, DLS measurements were taken under the concentration of 0.5 mg/mL.

### Pharmacokinetics and Bio-Distribution Studies

Four male rhesus macaques were single administered with 5 mg/kg of RTX and n-RTX via intravenous infusion in 30 mL sterile saline through femoral vein. 2–3 mL peripheral blood was collected in EDTA anticoagulant tubes and centrifuged at 5000 rpm/min for 2 min, the plasma was separated and freeze down in −80°C for further purposes. 300–500 μL CSF with no apparent blood contamination continually collected through 3–4th lumbar spine by 1 mL syringe and frozen in −80°C for ELISA test. All animals were anesthetized with an intraperitoneal injection of FFM mix (2.5 mg Fluanisone, 0.105 mg Fentanylcitrate, and 1.25 mg Midozalam HCl/kg in distilled water). On Days 21 and 64 post-injection, animals were euthanized; tissues were removed following heart perfusion with ice-cold saline and fixed in 4% paraformaldehyde for further analyses.

### Flow Cytometry

Five hundred microliters peripheral blood was used for staining following the plasma separation. Cells was rinsed by cold PBS (pH7.4) once, counted, and then suspended in FACS buffer (2% FBS/PBS), followed by blocking with 2 μL Human Trustain FcX (BioLegend, US) at room temperature for 10 min. Following the staining of dead cells with LIVE/DEAD Fixable Violet Dead cell staining kit, cells were incubated with PerCP-CY5.5-conjugated mouse anti-human CD3, FITC-conjugated mouse anti-human CD8, BV605-conjugated mouse anti-human CD4, PE-conjugated mouse anti-human CD19, APC-CY7-conjugated mouse anti-human CD20 in dark at 4°C for 30 min. Cells were then rinsed with PBS for two times and fixed by 2% paraformaldehyde in PBS. Expression levels were assessed by BD LSRFortessa™ (BD Biosciences, Inc.), and analyzed with FlowJo (FlowJo, LLC).

### Preparation of Fluorescence Labeled RTX

Rhodamine-B-labeled RTX was prepared by following the protocol provided by the manufacturer of fluorescence dyes. Fluorescent dyes, Rhodamine-B (RhB), were first dissolved in anhydrous DMSO to get 10 mg/mL stock solution, respectively. Then 50 μL of dye solutions were added gradually into 2 mL enzyme solutions (10 mg protein/mL, pH = 8.2, sodium carbonate, 100 mM). The reactions were carried out overnight at 4°C. Labeled RTX were then dialyzed against phosphate buffer (20 mM, pH = 7), condensed by centrifugal filtration (MWCO = 10 kDa) and stored at 4°C for further use. The concentration and dye/mAb ratio (D/P) were determined by the extinction coefficients of 2,101,000 M^−1^ cm^−1^ at 280 nm (RTX) and 108,000 M^−1^ cm^−1^ at 555 nm (RhB).

### Tissue Imaging

The bioluminescence imaging of organs was performed with IVIS Spectrum imager (PerkinElmer, Waltham, MA). Rats were injected through IV with 15 mg/kg of fluorescent labeled native RTX or n-RTX and scarified at Day 7 post-injection. The tissue images present the total photon flux *per second* within each organ with rainbow color scales. To further quantifiably compare the fluorescent intensity, and the fluorescent intensity from tissue homogenates was quantified as Relative Fluorescence Unit (RFU) per g.

### Immunohistochemistry

Axillary, mesenteric, and inguinal lymph nodes, frontal, parietal, temporal, occipital lobes, and cerebellum, were collected separately and fixed in formaldehyde. Four micrometers thickness section were cut serially post paraffin embedding. For staining, slides were first heated at 60°C for 1 h, then deparaffinized in xylene twice and rehydrated in an ethanol gradient. For antigen retrieval, LNs and brain lobes were treated with citrate buffer (Vector Laboratories and Biocare Medical, respectively) for 25 min at 100°C and for 50 min in pressure cooker post ddH_2_O rinse, respectively. Sections were incubated with BLOXALL endogenous peroxidase (Vector Laboratories) and alkaline phosphatase blocking solution (Vector Laboratories) for 10 min at room temperature, followed with PBST (0.1% Tween-20) wash and serum blocking at room temperature for 1 h. Primary mouse anti-human CD20 (1:200, clone L26, Santa Cruz Biotechnology), which recognizes different epitopes on rhesus CD20 molecules not covered by RTX to minimize CD20 epitope masking by rituximab, rabbit anti-human CD3 (1:100, cloneSP7, Invitrogen), and mouse anti-human Bcl-6 (1:100, cloneD8, Santa Cruz Biotechnology), were incubated overnight at 4°C, separately. Rabbit anti-human glia fibrillary acidic protein (GFAP) (1:200, Biocare Medical), mouse anti-human ionized calcium-binding adapter molecule 1 (IBA-1) (1:100, clone 20A12.1, Millipore) were separately used to identify astrocyte and microglia, respectively in brain lobes. After rinsed in PBST, the slides were incubated with ImmPRESS HRP universal secondary antibody at room temperature for 30 min. Followed with PBST wash, 3,3′-diaminobenzidine (DAB) staining, nucleus counterstaining, graded ethanol dehydration, xylene clear, the sections were covered with mounting medium (Thermo Fisher). Images were captured by inverted microscope (DMi1, Leica), brown staining was considered as positive signal, otherwise was considered as negative. Slides of lymph nodes were analyzed with Image J (NIH); slides of brain lobes were scanned by Aperio ScanScope slide scanner by 4 × 4 grid (20×).

For analysis of lymph nodes, 6.8 × 5 cm of each 40 magnified CD20 and CD3 positive staining (*n* = 7) and 3.4 × 4.2 cm of Bcl6 (*n* = 4), was randomly selected and their optical density (OD) numbers were calculated with the following formula: OD = log (max intensity/Mean intensity), where max intensity = 255 for 8-bit images. Brain slides were performed with the Aperio Positive Pixel Count v9 algorithm. Positive pixel calculation for GFAP used the following variables: hue width = 0.371 and color saturation threshold = 9.7e-002. Positive pixel calculation for IBA-1 used the following variables: hue width = 0.5 and color saturation threshold = 4.0e-007. Positivity was calculated by total number of positive pixels divided by the total number of pixels. The value was then multiplied by 100 to give a percentage for positive pixels. Total area analyzed for both GFAP and IBA is 4 mm^2^. One-way ANOVA from SPSS software (IBM) was used for the analysis of statistical significance (^**^p < 0.01), the inversed gray value of positive stained tissue was showed in histogram (mean ± SD).

### MAb Detection by Enzyme-Linked Immunosorbent Assays (ELISA)

The concentration of RTX in animal body fluids and tissue homogenates was measured by ELISA against RTX. RTX levels were measured by ELISA using a monoclonal antibody (HCA062, clone#AbD02844, Bio-Rad, Hercules, CA), which specifically recognizes the idiotypic determinants of RTX. The 96-well plates were coated with 1 μg/mL of anti-RTX antibody (diluted in sodium carbonate–bicarbonate buffer), followed by blocking with 1% BSA/PBS for 2 h at room temperature. Diluted samples of RTX in PBST from 0 to 500 ng/mL were then added and incubated for 1 h at room temperature to obtain calibration curves. Animal body fluids and tissue homogenates containing encapsulated RTX in non-degradable nanocapsules were treated with 100 mM sodium acetate buffer (pH 5.4) at 4°C overnight and used for ELISA measurement. Released RTX from hydrolysable nanocapsules was directly measured with animal body fluids and tissue homogenates. All animal samples were added and incubated for an additional hour at room temperature. After washing with PBST for five times, peroxidase-conjugated anti-human Fc antibody was added and incubated for a further hour at room temperature. The substrate 3,3′,5,5′-Tetramethylbenzidine (TMB) solution was added and incubated until the appropriate color developed. The reaction was stopped and absorbance at 450 nm was measured with a plate reader (FLUOstar OPTIMA).

### Animal Care and Ethics Statements

All research involving animals was conducted according to relevant national and international guidelines. Male SD rats (weighting 180–220 g, 6–8 weeks old) were provided by the Experimental Animal Center of Medical Department of Peking University. All interventions and animal care procedures were performed in accordance with the Guidelines and Policies for Anima l Surgery provided by our collaborator institute (Chinese Academy of Medical Sciences & Peking Union Medical College, Beijing, China) and were approved by the Institutional Animal Use and Care Committee. The rats were maintained in a temperature-controlled facility (temperature: 22 ± 1°C, humidity: 60%) with a 14 h light/10 h dark photoperiod and free access to food and water. Male rhesus macaques (weighing 4.8–5.2 kg, 4–5 years old) were purchased from the Medical Primate Research Center of the Institute of Medical Biology, Chinese Academy of Medical Sciences, and housed and bred according to the guidelines. The experimental protocols were reviewed and approved by the Yunnan Province Experimental Animal Management Association (SYXK-YN 2010-0009) and the Experimental Animal Ethic Committee of the Institute, which complied with the humane regulations of replacement, refinement, and reduction.

## Data Availability Statement

The datasets generated for this study are available on request to the corresponding author.

## Ethics Statement

The animal study was reviewed and approved by 1. Institutional Animal Use and Care Committee, Chinese Academy of Medical Sciences and Peking Union Medical College, Beijing, China. 2. Yunnan Province Experimental Animal Management Association (SYXK-YN 2010-0009) and the Experimental Animal Ethic Committee of the Institute.

## Author Contributions

MQ, MK, and JW designed studies. MQ, LW, DW, CW, DX, QG, JG, EK, YLuo, YLee, and YX performed experiments. HV, GS, XS, ZH, YLu, MK, JW, and IC discussed and interpreted data. MQ, JW, MK, and IC wrote the paper.

### Conflict of Interest

IC has a financial interest in CSL Behring and Calimmune Inc. No funding was provided by these companies to support this work. The remaining authors declare that the research was conducted in the absence of any commercial or financial relationships that could be construed as a potential conflict of interest.
