# Supplementary material for: Enhanced Delivery of Rituximab Into Brain and Lymph Nodes Using Timed-Release Nanocapsules in Non-Human Primates
Source: Front Immunol. 2020 Jan 23;10:3132. doi: 10.3389/fimmu.2019.03132 (PMC6996053; doi:10.3389/fimmu.2019.03132)
Supplement: Supplementary file 1 [file Data_Sheet_1.pdf]

Supplementary Information

Fig. S1. Increased biodistribution of Herceptin (anti-Her2) by nanocapsules in mouse CNS.

Fig.S2. Nanocapsulated RTX treatment does not affect blood chemistry values in rhesus macaques.

Fig.S3. Nanocapsulated RTX treatment does not change absolute counts of each blood cell in rhesus macaques.

Fig.S4. Nanocapsulated RTX mediates superior B-cell depletion in LNs of rhesus macaques confirmed by flow cytometry.

Table S1. Synthesis formulation of nanocapsules.

Fig. S1. Increased biodistribution of Herceptin (anti-Her2) in mouse CNS. Biodistribution of Herceptin (anti-Her2) and Herceptin nanocapsules (n-Herceptin) in mice. The concentration of native (Herceptin) and encapsulated Herceptin (n-Herceptin) in mouse body fluids and homogenates of perfused brain was measured by ELISA at day 1 after IV injection of 500µg Herceptin. Data were generated from n=4 mice. Data are shown as means ± s.d. of biological quadruplicate. Statistical significance, compared with the native group, was determined by Mann–Whitney–Wilcoxon test. *: significant, $p<0.1$.

Fig.S2. Nanocapsulated RTX treatment does not affect blood chemistry values in rhesus macaques. Whole blood was collected from rhesus macaques after the single administration with 5mg/kg native RTX or n-RTX as scheduled. Plasma fractions were separated after centrifugation at 3000rpm for 10 min and served for blood chemistry test including a panel for serum globulin (GLB), indirect bilirubin (IBIL), gamma-glutamyl transferase (γ-GT), total protein (TP), albumin (ALB), hemoglobin (HGB), hematocrit (HCT), and platelet count (PLT).

Fig.S3. Nanocapsulated RTX treatment does not change absolute counts of each blood cell in rhesus macaques. The whole blood was collected from rhesus macaques after the single administration with 5mg/kg native RTX or n-RTX as scheduled. Absolute numbers of each blood cell type were quantified by blood routine counter (Sysmex XT-2000iV) and plotted for white blood cells (WBC), lymphocytes, monocytes and neutrophils.

Fig.S4. Nanocapsulated RTX mediates superior B-cell depletion in LNs of rhesus macaques confirmed by flow cytometry. Inguinal LNs were collected by biopsy before and 3, 10, 17 days after the infusion, and at necropsy on Day21 from Group I rhesus macaques with a single dose (5mg/kg) of native RTX or n-RTX via IV and processed to get single cells. B cell were stained with CD19 and CD20, while T cells were stained by CD3. The B and T cell percentages were checked by flow cytometry.

Table S1. Synthesis parameters of degradable nanocapsules.

Sample	Protein	MPC	GDMA	AI102	APS	TEMED
**n(RTX)**	1	12000	500	500	2000	4000
