# Supplementary figures and images for: Enhanced Delivery of Rituximab Into Brain and Lymph Nodes Using Timed-Release Nanocapsules in Non-Human Primates
Source: Front Immunol. 2020 Jan 23;10:3132. doi: 10.3389/fimmu.2019.03132 (PMC6996053; doi:10.3389/fimmu.2019.03132)

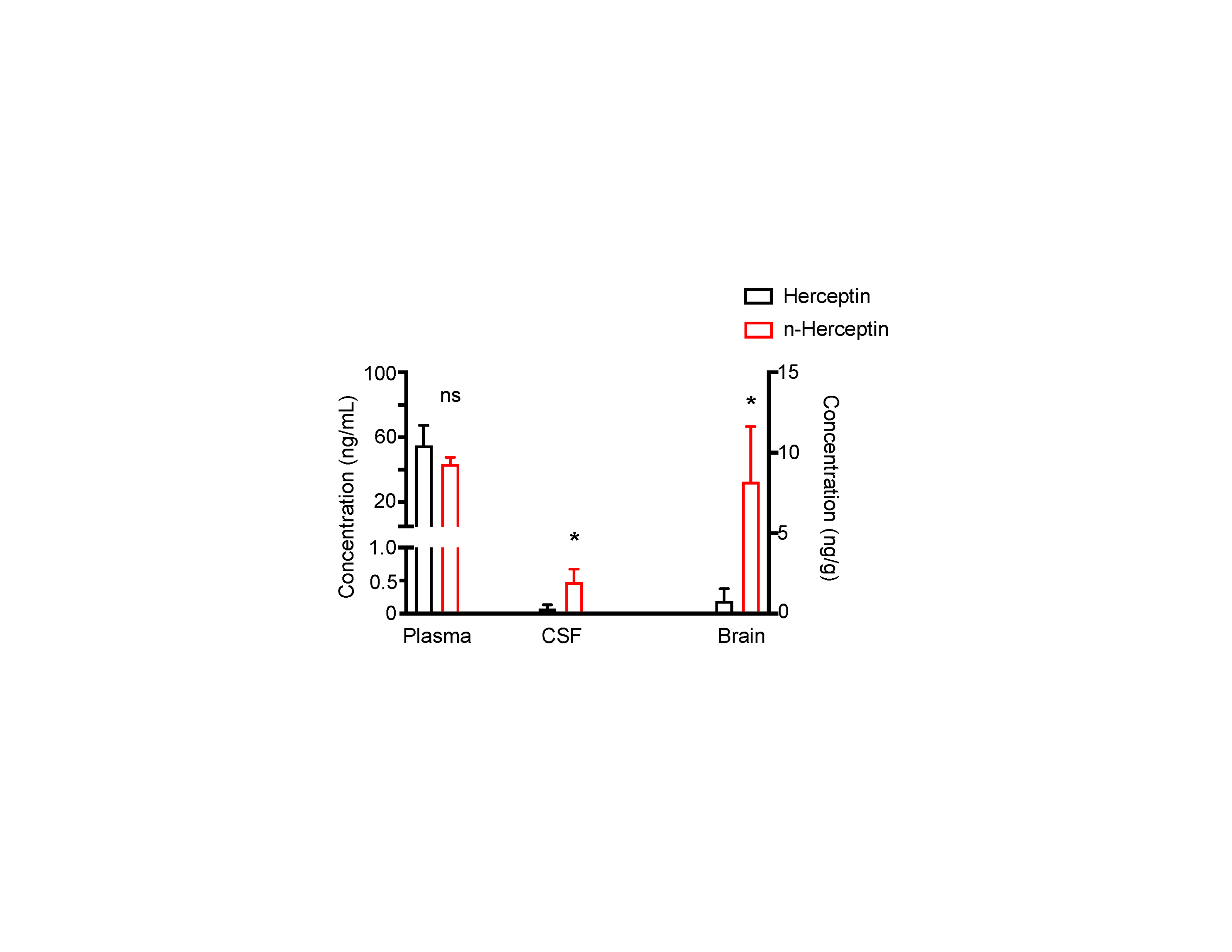

Supplement: Supplementary file 2 [file Image_1.tif]

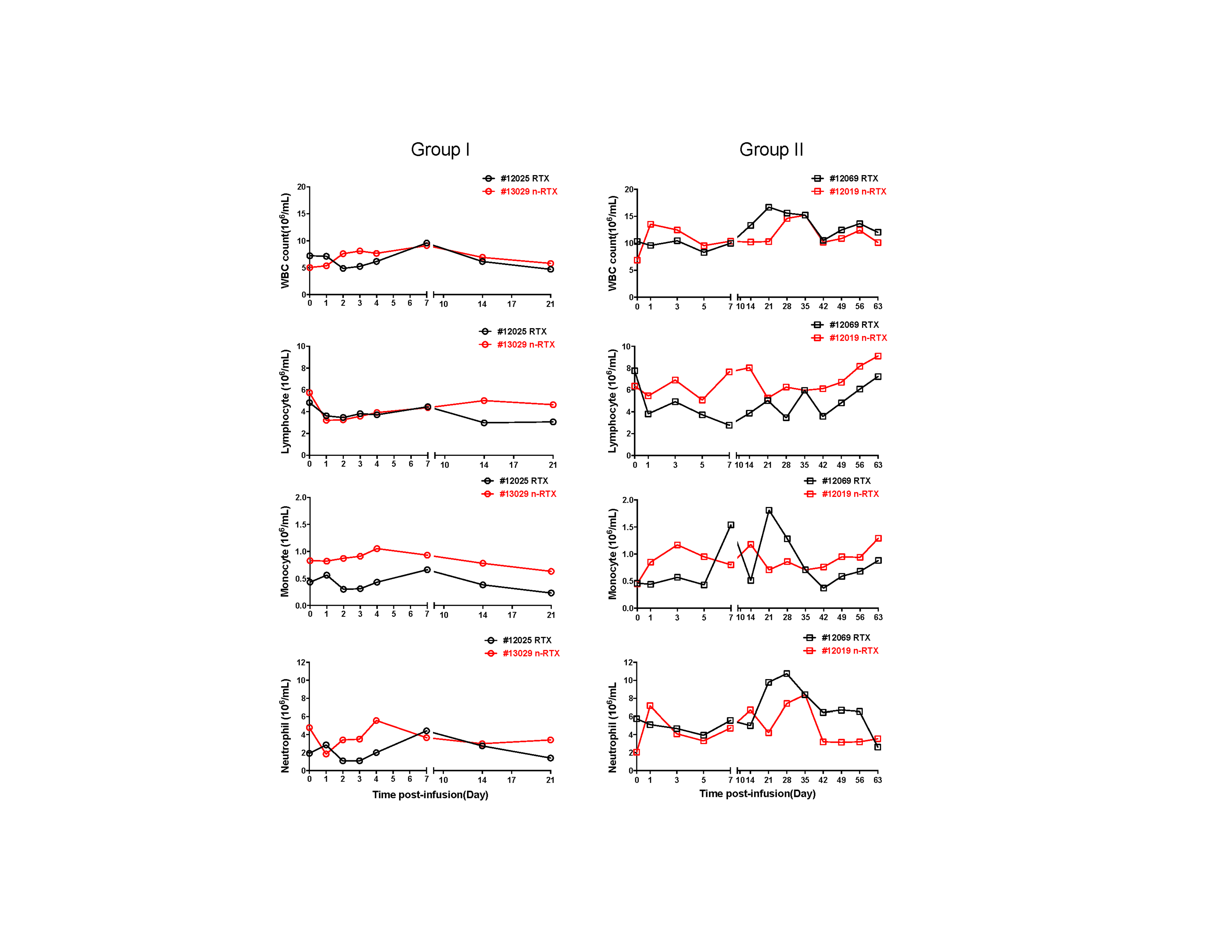

Supplement: Supplementary file 3 [file Image_2.tif]

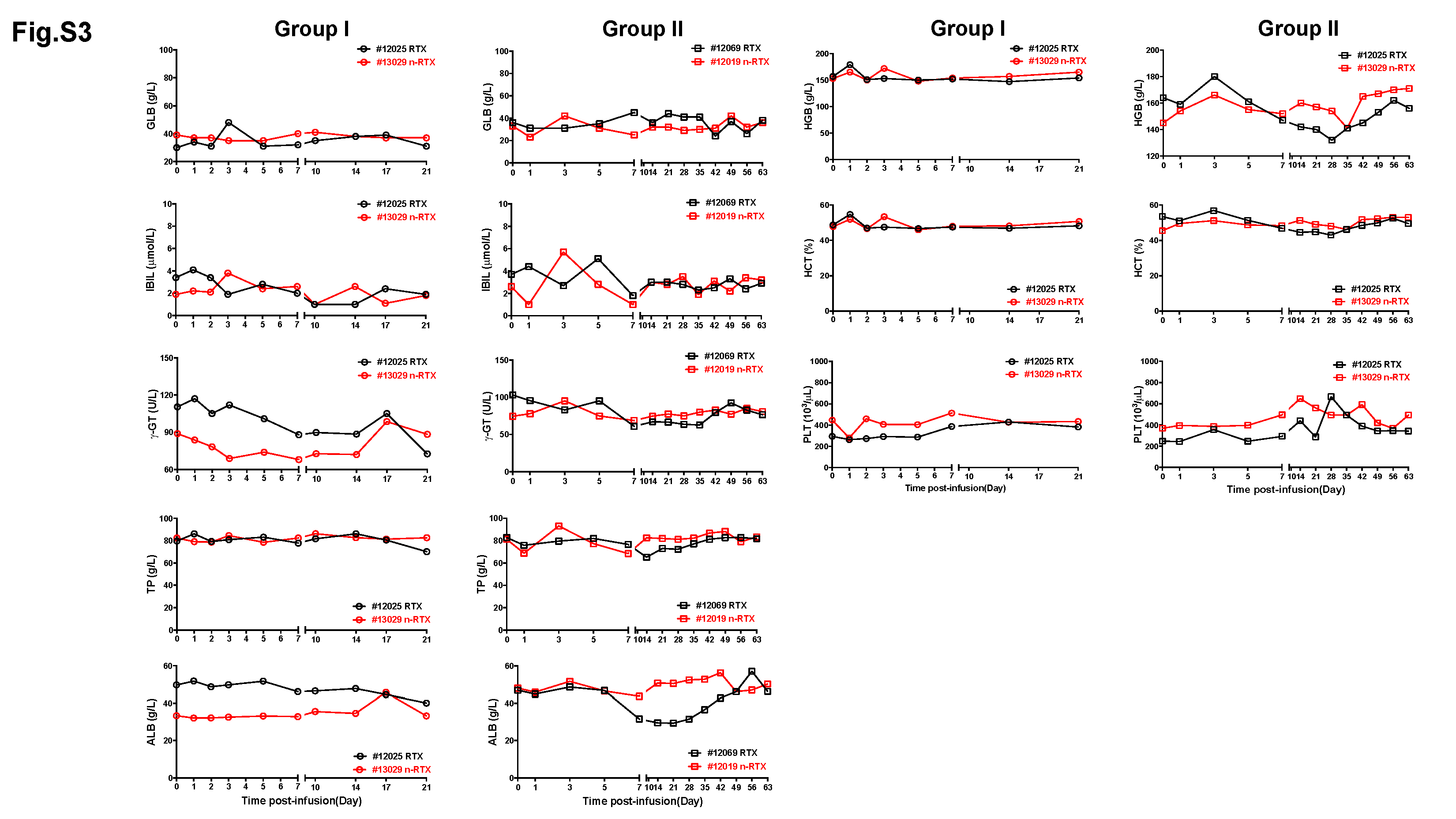

Supplement: Supplementary file 4 [file Image_3.tiff]

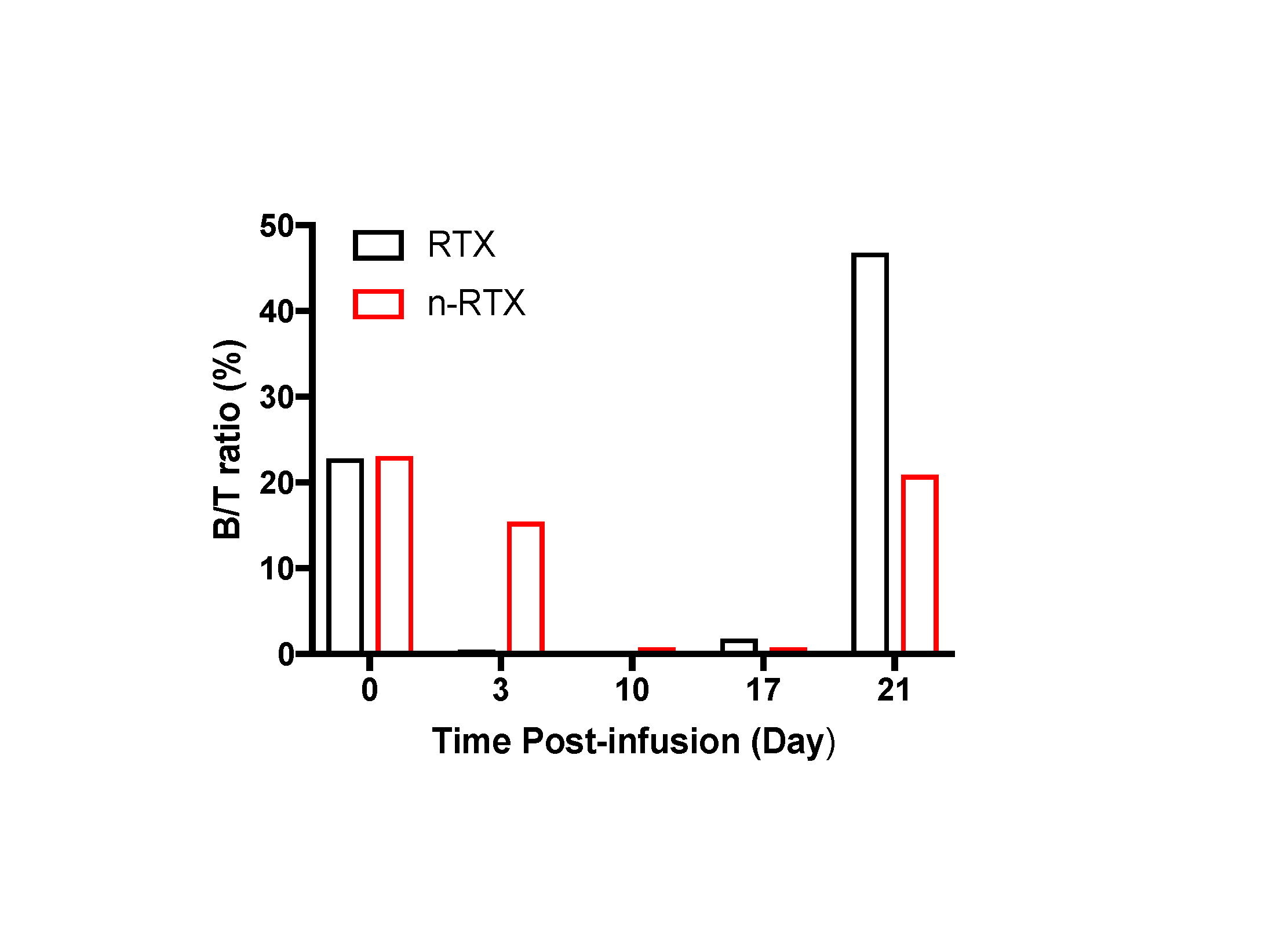

Supplement: Supplementary file 5 [file Image_4.tif]
